# Effect of myrtle fruit syrup on abnormal uterine bleeding: a randomized double-blind, placebo-controlled pilot study

**DOI:** 10.1186/2008-2231-22-45

**Published:** 2014-06-02

**Authors:** Marzieh Qaraaty, Seyed Hamid Kamali, Fataneh Hashem Dabaghian, Nafiseh Zafarghandi, Roshanak Mokaberinejad, Masumeh Mobli, Gholamreza Amin, Mohsen Naseri, Mohammad Kamalinejad, Mohsen Amin, Azizeh Ghaseminejad, Seyedeh jihan HosseiniKhabiri, Daryush Talei

**Affiliations:** 1 Traditional Medicine Clinical Trial Research Center, Shahed University, Tehran, Iran; 2 Department of Traditional Medicine, Faculty of Traditional Medicine, Shahid Beheshti University of Medical Sciences, Tehran, Iran; 3 Research Institute for Islamic and Complementary Medicine, Iran University of Medical Sciences, Tehran, Iran; 4 Department of Gynecology and Obstetrics, Faculty of Medical Sciences, Shahed University, Tehran, Iran; 5 Department of Traditional Medicine, School of Traditional Medicine, Shahid Beheshti University of Medical Sciences, Tehran, Iran; 6 Department of Traditional Pharmacy, Faculty of Traditional Medicine, Tehran University of Medical Sciences, Tehran, Iran; 7 Department of Pharmacognosy, School of Pharmacy Shahid Beheshti University of Medical Sciences, Tehran, Iran; 8 Department of Drug and Food control, Faculty of Pharmacy, Tehran University of Medical Sciences, Tehran, Iran; 9 Department of Gynecology and Obstetrics, Tehran University of Medical Sciences, Tehran, Iran; 10 Khatam Hospital, Tehran, Iran; 11 Medicinal Plant Research Centre, Shahed University, Tehran, Iran

**Keywords:** Abnormal uterine bleeding-menometrorrhagia, Effrat-e-tams, Iranian traditional medicine, *Myrtus communis* L, Myrtle, Myrtaceae

## Abstract

**Background:**

Myrtle (*Myrtus communis* L.) has been used in the Iranian Traditional Medicine as a treatment for abnormal uterine bleeding-menometrorrhagia. The main aim of this study is to evaluate the effect of myrtle fruit syrup on abnormal uterine bleeding-menometrorrhagia.

**Methods:**

A randomized, double-blind, placebo-controlled pilot study was conducted on 30 women suffering from abnormal uterine bleeding-menometrorrhagia. Treatment comprised of giving 15 ml oral myrtle syrup daily (5 ml three times a day) for 7 days starting from the onset of bleeding. The myrtle syrup along with placebo was repeated for 3 consecutive menstrual periods. Menstrual duration and number of used pads were recorded by the Pictorial Blood loss Assessment Chart at the end of each menstrual period. The quality of life was also evaluated using the menorrhagia questionnaire.

**Results:**

The mean number of bleeding days significantly declined from 10.6 ± 2.7 days to 8.2 ± 1.9 days after 3 months treatment with the syrup (p = 0.01) and consequently the participants in the intervention group used fewer pads after 3 months (16.4 ± 10.7) compared with the number of pads used at the beginning of the treatment (22.7 ± 12.0, p = 0.01). Bleeding days and number of pads used by the participants in the placebo group did not change significantly. Also significant changes of quality of life scores were observed in the intervention group after 3 months compared to the baseline.

**Conclusion:**

Myrtle syrup is introduced as a potential remedy for abnormal uterine bleeding-menometrorrhagia.

## Introduction

Abnormal uterine bleeding (AUB) is one of the main reasons of visiting gynecologists [1]. AUB affects up to one-third of sexually active women [2] and the overall prevalence of this abnormality is 11%-13%, reaching 24% at the age of 36–40 [3]. AUB has a considerable high morbidity rate among women of childbearing age and imposes major medical, social and financial burdens on women, their families and health services [4]. Different types of AUB include a range of dysfunctional conditions affecting regularity, frequency, duration or volume of menstrual flow [5,6]. Menorrhagia or hypermenorrhea is defined as menstrual blood loss of more than 80 ml per cycle or longer than 7 days or both of them [7], while polymenorrhea is defined as having menstruations about every 21 days and occasionally at even shorter intervals causing irregular ovulation. Metrorrhagia is uterine bleeding at irregular intervals, particularly between the expected menstrual periods [8]. Abnormal uterine bleeding-Menometrorrhagia (AUB-MM) is defined as prolonged and excessive uterine bleeding in irregular intervals [9]. The most common causes of AUB may be pregnancy, genital tract diseases, certain medical conditions such as thyroid dysfunctions and hypothalamic suppressions including stress, weight loss, excessive exercise, and even coagulopathies [1,10]. AUB treatment includes administration of non-steroidal anti-inflammatory drugs (NSAIDs), antifibrinolytics such as tranexamic acid, cyclic oral progestins, oral contraceptives and levonorgestrel-releasing intra-uterine system [1,5,11]. Hormone therapies have many side effects [12] and the common complication of tranexamic acid is gastrointestinal disturbances [13]. AUB involves two-thirds of all hysterectomies leading to several complications [14,15].

Iranian Traditional Medicine (ITM) practitioners such as Ibn Sina (Avicenna, 980–1037 A.D) believed that the normal menstruation is a good sign of healthy status of a woman which results in chastity and modesty [16-18]. In ITM literature, AUB is described under the title of “Effrat-e-Tams” or “Kasrat-e-Tams” [7,18]. Menometrorrhagia is more compatible with Effrat-e-Tams in ITM [17-20].

Based on ITM literature, particularly Avicenna’s book (Al-Qanun fit-teb or Canon of medicine, 1025 A.D), myrtle is known as “*mourd*” or “aa*ss*”  and its fruit that called *Habbol- aass*, is one of the effective medicinal herbs for decreasing the menstrual bleeding [18]. Myrtle is a fragrant evergreen shrub belonging to *myrtaceae* family, growing wild in Iran [21,22] and the Mediterranean area. The fruits have sweet-spicy tastes that are very astringent [23]. Myrtle has been used as antiviral, antifungal, antiseptic and antioxidant agent [24,25]. Myrtle berries extract has ulcer-protective properties [26] and anti-inflammatory effects [27]. The essential oils obtained from leaves, flowers and fruits have been used in flavor and fragrance industries [28]. Its biological effect in menstrual disturbances has been described in ITM which may be novel in modern medicine [18].

There is a lack of detailed trials on the effects of myrtle syrup on menstruation. The main objective of the present study was to investigate the effects of myrtle syrup on reducing AUB-MM in a pilot placebo-controlled clinical trial.

## Materials and methods

### Study design and target group

**Figure 1:**
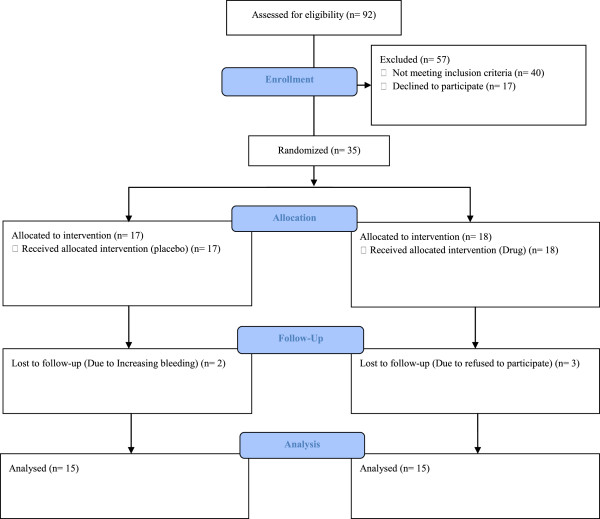
Study flow chart.

In this randomized, double-blinded, placebo-controlled pilot study, 30 patients were randomly assigned into two groups of placebo (n = 15) and myrtle treatment (n = 15). Participants were treated with either 15 ml of myrtle fruit syrup or placebo, 3 times a day for seven days starting from the onset of bleeding. The treatment was performed for 3 consecutive menstrual periods. Randomization of equal number of subjects to placebo or treated group was achieved using a simple random allocation strategy, using block randomization method. The participants were selected according to the defined inclusion criteria: 20 to 55 years old, married women, not disposed toward hormone therapy, not pregnant, not lactating, normal gynecological observations, normal pap smear, endometrial thickness less than 12 mm, menstrual period more than 7 days in duration and/or less than 21 days from the start of one period until the start of the next menstrual period and/or clot excretion, use of more than 10 sanitary product items in a cycle. Sexually active women were required to use a suitable non-hormonal birth control. Initially, 92 patients were interviewed from which, 35 patients were recruited and randomized in two groups of placebo and extract treatment. 30 participants completed the study, 15 in each group (Figure 1). Two participants in the placebo group discontinued their therapy because of increasing bleeding during first cycle. One subject in the intervention group did not use the syrup completely and two persons discontinued the study because of personal reasons.

Women were excluded from the study if they had a history of significant medical problems (coagulopathies, diabetes mellitus, chronic inflammatory disease, thyroid dysfunctions); had a history of endometrial abnormalities (such as hyperplasia), cervical carcinoma, uterine or ovary malignancy; sub-mucosal or intramural fibroids more than 5 cm; needed surgery and emergency procedure because of increasing bleeding during the study. All of the subjects were free to withdraw at any time during the course of study.

Participants were not permitted to use mefenamic acid, tranexamic acid, any hormonal therapy, herbal medicine and medicinal herb during the study. Use of acetaminophen, oral iron therapy and analgesic opioids was permitted throughout the study.

All participants signed a written informed consent before recruiting in the study. The Ethics Committee of Shahed University approved the protocol (approval number: 41/138342). In addition, the trial was registered in the Iranian Registry of Clinical Trials under the number IRCT 201109077511 N1.

### Plant material

Myrtle dried berries were collected from Manjil on road to Gilan (North of Iran) in 2011 and its identity was authenticated by Professor Gholamreza Amin. A voucher specimen of the plant has been deposited in Herbarium Tehran University of Medical Sciences, Faculty of Pharmacy under the voucher No 6632-TEH.

### Preparation of syrup and placebo

Traditional decoction was prepared as described in “Qarabadin” (Ghayeni, Qarabadin-e-Salehi, 1765 AD; Aghili, Qarabadin-e-Kabir, 1781 AD) [29,30] texts belonging to ITM pharmaceutical discipline. 63 g of the pulverized samples of myrtle fruits were macerated for 24 hours with 200 ml of distilled water, filtered and boiled for 15 min. 108 g sucrose was added to the extract in order to prepare the syrup. The medication was supplied in bottles of 120 ml, containing either drug or placebo.

Placebo was prepared based on pharmacopoeia simple syrup formula including approved color additives and looked the same as the myrtle syrup.

Myrtle syrup is standardized based on total phenols (Folin-Ciocalteau method) and gallic acid (Rhodanine assay) content. Each 5 ml of syrup contains 0.05 ± 0.03 g dry residue and 41 mg total phenols as gallic acid equivalents.

The participants were given either 5 ml of prepared syrup or placebo three times a day, 30 minute after each meal for seven days starting from the onset of bleeding. This treatment was repeated for three consecutive menstruation cycle.

The myrtle syrup and placebo were identical in the same physical form, packaging and labeling and divided to groups 1 and 2. Physician prescribed syrups to the patients according to the label numbers. Physician and presenter of the myrtle syrup or placebo were blind for the contents. The pharmacist was the only person who was aware of the numbers assigned to the myrtle syrup or placebo.

### Bleeding measurements

All the participants were evaluated based on a complete medical history and gynecological examination. Menstrual blood loss was assessed with Pictorial Blood loss Assessment Chart (PBAC). The quality of life was evaluated with menorrhagia questionnaire (MQ-Iranian Version) [31,32] before treatment and at the end of the study. Certain blood test including complete blood count (CBC), prothrombin time (PT), partial thromboplastin time (PTT), follicle-stimulating hormone (FSH), luteinizing hormone (LH) and thyroid stimulating hormone (TSH) were done before the study. PT and PTT were done to exclude bleeding disorders. TSH, FSH and LH were done to exclude thyroid dysfunction and hypothalamic pituitary dysfunction, respectively. CBC was performed to determine hemoglobin (Hb) and hematocrit (Hct). Trans-vaginal ultra sonography was also performed to find out if the subject had any pelvic pathological disorders and to determine the endometrial thickness. Cervical cytology (Pap smear) was done to rule out other abnormalities.

Menstrual blood loss and menstrual duration were measured using PBAC chart during three consecutive treatment cycles and was compared with the ones at the beginning of the treatment (baseline). The participants were requested to report the details of their menstrual cycle i.e., the start date, duration of menstruation, the number of sanitary pads used (considered as the intensity of bleeding) and any adverse effects. The information was recorded at the beginning of the treatment and at the end of each menstrual cycle. The PBAC chart had a sensitivity of 80% and specificity of 88% in diagnosing menorrhagia (as defined in the alkaline hematin method) [33].

### Statistical analysis

The primary outcome measures included the duration of menstrual period, number of pads used during menstruation. The MQ score and the side effects were the secondary outcome measures.

Normal probability plot was used to test for normality of data in GraphPad Prism version 5. The data points appeared linear on the plot and the data were considered as normal distribution. Repeated-measures ANOVA function in the program GraphPad Prism version 5 was used to test for differences of primary outcomes within the groups. Repeated-measures ANOVA compares the means of more than two matched groups in a longitudinal study in which change over time is assessed. Student’s *t*-test was used to compare the MQ scores before and after the treatment.

## Results

### Baseline characteristics

The baseline characteristics of the subjects are described on Table 1. There were no statistically significant differences in baseline characteristics between the groups. Hence, the groups were homogenous with respect to age, level of education and investigations. Age of the patients ranged from 20 to 55 years with the mean age 41.2 ± 6.9 years.

**Table 1:** Baseline characteristics of study subjects

**Parameter**	**Intervention group**	**Placebo group**	**P value**
Age	41.33 ± 7.228	41.13 ± 6.978	0.5
BMI	28.86 ± 4.68	31.99 ± 6.29	0.2
MQ score	47.8 ± 15.7	41.2 ± 15.3	0.2
Duration of abnormality (month)	53.93 ± 61.46	72.33 ± 69.92	0.3

### Effects of myrtle fruit syrup on duration and intensity of bleeding

The average number of bleeding days and number of pads used during the study are summarized on Table 2. There was not statistically significant difference between the groups in terms of bleeding days and number of pads at the beginning of the study.

**Table 2:** The effect of myrtle fruit syrup in bleeding at baseline and post treatment

**Variable**	**Group**	**Title**	**Mean(±SD)**	**Mean difference (±SE) compared with baseline**	95% CI*	P values**
Menstrual duration (day)	Intervention (n = 15)	Baseline	10.6(2.7)			
		After 1^st^ cycle	8.8(2.3)	1.7(0.6)	-0.3- 3.8	0.08
		After 2^nd^ cycle	8.9(3.8)	1.6(0.9)	-1.3- 4.7	0.08
		After 3^rd^ cycle	8.2(1.9)	2.3(0.6)	0.3- 4.3	0.01
	Placebo (n = 15)	Baseline	9.8(3.5)			
		After 1^st^ cycle	8.8(3.2)	1(0.5)	-0.7- 2.7	0.6
		After 2^nd^ cycle	8.7(2.6)	1.1(0.6)	-0.8- 3.1	0.5
		After 3^rd^ cycle	8.6(3.2)	1.2(0.4)	-0.08- 2.6	0.5
Number of pads used	Intervention (n = 15)	Baseline	22.7(12)			
		After 1^st^ cycle	20(14)	2.6(1.6)	-2.4--7.7	0.5
		After 2^nd^ cycle	21.4(17.9)	1.3(2.7)	-7.1- 9.7	0.8
		After 3^rd^ cycle	16.4(10.7)	6.3(1.5)	1.6- 11	0.01
	Placebo (n = 15)	Baseline	15.4(9.8)			
		After 1^st^ cycle	13.9(7.2)	1.5(1.7)	-3.7- 6.8	0.6
		After 2^nd^ cycle	11.6(7.8)	3.8(1.5)	-0.7- 8.4	01
		After 3^rd^ cycle	15(8.7)	0.4(2)	-5.9- 6.7	0.9
MQ score	Intervention (n = 15)	Baseline	47.8(15.7)			
		After 3 months	39.4(16.7)	8.4(3.7)	0.4-16.4	0.02
	Placebo (n = 15)	Baseline	41.2(15.3)			
		After 3 months	39.2(14.5)	2(2.03)	-2.3- 6.4	0.7

*One-way analysis of variance (ANOVA) was used to compare the groups before and after each treatment with either placebo or extract. There was statistically significant difference between groups before and after three rounds of treatment with myrtle syrup, while the difference between groups before and after placebo treatment was not statistically significant.

**P values <0.05 are significant.

The number of bleeding days and consequently number of pads used by the participants significantly decreased in the intervention group after 3 months (P = 0.01), while changes of these variables were not significant in the placebo group.

Significant changes of MQ score was observed in the intervention group after 3 months compared to the baseline (P = 0.02).

## Discussion

To the best of our knowledge, the present study is the first randomized placebo-controlled trial on the effects of myrtle fruit in women with AUB-MM. The results of this study showed that myrtle syrup had notable advantages over placebo in women with AUB-MM. Also, the quality of life was significantly improved in the intervention group with minor side effects.

During luteal phase in menstrual cycle, some inflammatory processes lead to tissue edema in endometrium and continue with excessive menstrual bleeding (EMB). Unusual secretion of local pro-inflammatory cytokines responsible in the vascular tone has been observed [2,4]. In these women, endometrium synthesizes much more prostaglandin E_2_ (PGE_2_) than it does with vasoconstrictor PGF_2α_. A noticeable increased PGE_2/_PGF_2α_ ratio happens during luteal phase in women with menstrual blood loss (>90 ml). Endometrial synthesis of PGs and signaling in women with profuse menstruation is greater than women with normal menstrual bleeding [4]. These inflammatory molecules can be targeted to treat the disturbances in women suffering from AUB.

Myrtle berries aqueous extract contains phenolic-like tannins (galllic acid derivatives), anthocyanins and flavonoids [34]. Tannin-containing medications have been used traditionally as styptics [35]. Anti-inflammatory activities of anthocyanins have been proven in some studies [36]. Some studies have demonstrated that flavonoids can inhibit inflammatory mediators [37]. According to a preliminary study, micronized flavonoids suppressed endometrial prostaglandins and were safe and effective in AUB [38]. Another phytochemical compound in myrtle that suppresses prostaglandin E_2_ formation efficiently is myrtucommulon [39]. Therefore, the presence of the effective anti-inflammatory components in the myrtle extract can render the myrtle syrup a potential source to reduce prostaglandin secretion and to cure AUB subsequently. Further mechanistic studies are suggested to prove the anti-inflammatory effects of the components in the myrtle extract.

This study had some potential limitations which are usually part of the nature of human studies. Firstly, ITM has two groups of principal variables: one is part of human nature, *mezaj* (temperament), racial/ethnic, sex, age, season, zone, profession [40], and the second factor is the composition of the herbal preparations which may vary based on the geographical habitat of the plant, the climate, and the time of reaping [41]. These factors have not been considered in our study.

In the present study, the subjects received syrup only for three cycles; therefore we cannot comment on any long-term efficacy of myrtle syrup. Also, the subjects were not followed up after finishing the study and the long-lasting effects are not clear to us.

## Conclusion

The outcomes of this study showed that myrtle syrup is an effective drug as a short-term treatment of AUB-MM. Women in the test group experienced significant reductions of bleeding duration, as well as a significant decline of the intensity of bleeding while placebo did not affect the variables significantly. The quality of life improved among the subjects in the syrup-treated group. Based on the current novel results, a therapeutic role of myrtle syrup is suggested for women with AUB-MM, which is accessible and cost-effective therapy. Larger and longer randomized trials are being planned in our research group to confirm the long-term effects of myrtle on bleeding reduction in AUB-MM.

## Abbreviations

AUB-MM: Abnormal uterine bleeding-menometrorrhagia; CBC: Complete blood count; EMB: Excessive menstrual bleeding; FSH: Follicle- stimulating hormone; Hb: Hemoglobin; Hct: Hematocrit; HMB: Heavy menstrual bleeding; ITM: Iranian traditional medicine; LH: Luteinizing hormone; MQ: Menorrhagia questionnaire; PG: Prostaglandin; PT: Prothrombin time; PTT: Partial thomboplastin time; TSH: Thyroid stimulating hormone.

## Competing interests

The authors do not have any financial/ commercial competing interest in the study presented here.

## Authors’ contributions

MQ has made substantial contribution in designing, acquisition of data, and drafting the manuscript and has given the final approval of the version to be published. SHK participated involved in design, and revising, have given the final approval of the version to be published. FHD analyzed and interpreted the data. NZ the supervisor of conduction of the study, participated involved in design, and revising, have given the final approval of the version to be published. RM participated involved in revising. MM participated involved in revising. GHA participated in the identification of the plants, plant extraction and made substantial contributions in the study. MN co- study designer. MK participated involved in revising. MA participated involved in revising and analyzing the data of the manuscript. AGH participated involved in recruitment. SJHK participated involved in randomization procedure. DT participated involved in revising. All authors read and approved the final manuscript.
